# Chromosome-Level Genome and Variation Map of Eri Silkworm *Samia cynthia ricini*

**DOI:** 10.3390/biology14060698

**Published:** 2025-06-14

**Authors:** Kunpeng Lu, Jianghong Shen, Wengong Huang, Chengyu Zhan, Zhengqing Li, Shubo Liang, Kerui Lai, Qun Luo, Minjin Han, Xiaoling Tong, Fangyin Dai

**Affiliations:** 1 State Key Laboratory of Resource Insects, Key Laboratory of Sericultural Biology and Genetic Breeding, Ministry of Agriculture and Rural Affairs, College of Sericulture, Textile and Biomass Sciences, Southwest University, Chongqing 400715, China; lukunpeng@swu.edu.cn (K.L.); sjh0522@email.swu.edu.cn (J.S.); zhancyres@email.swu.edu.cn (C.Z.); lerche1999@email.swu.edu.cn (Z.L.); shuboliang@email.swu.edu.cn (S.L.); laijirong@outlook.com (K.L.); minjinhan@126.com (M.H.); xltong@swu.edu.cn (X.T.); 2 Guangxi Key Laboratory of Silkworm Genetic Improvement and Efficient Breeding, Guangxi Research Academy of Sericultural Science, Nanning 530007, China; gx_hwg@163.com (W.H.); 13507718201@163.com (Q.L.)

**Keywords:** eri silkworm, genome assembly, annotation, variation map, database

## Abstract

The eri silkworm *Samia cynthia ricini* (*S. ricini*) is an economically and scientifically significant lepidopteran species, though its genomic resources have remained limited. Here, we present a chromosome-level genome assembly for *S. ricini* generated through integrated long-read, short-read, and Hi-C sequencing data. The final 456.16 Mb assembly spans 14 chromosomes, exhibiting 98.5% BUSCO completeness and a 48.51% repetitive content. Functional annotation of the 15,729 protein-coding genes against five major databases (NR, SwissProt, Pfam, GO, and KEGG) revealed a maximum annotation rate of 92.71%, demonstrating high gene set quality. Comparative genomics with *B. mori* uncovered conserved syntenic blocks interspersed with chromosomal fusion/fission events and inversions. We further identified 4.27 million SNPs, 1.02 million InDels, and 53,367 SVs, establishing the first comprehensive variation map for this species. These genomic variations provide a foundation for marker-assisted breeding programs and trait association studies. All the genomic resources and interactive visualization tools were integrated into the SilkMeta database. This study establishes *S. ricini* as a pivotal resource for comparative lepidopteran genomics and accelerates molecular breeding programs for this agriculturally valuable insect.

## Simple Summary

The eri silkworm (*Samia cynthia ricini*) is a resource insect valued for its silk production, nutrient-rich pupae used in food and animal feed, and unique biological traits that intrigue scientists. Despite its importance, genomic resources for this species have remained limited. In this study, we generated a chromosome-level genome (456.16 Mb) using advanced DNA sequencing technologies, revealing key genomic insights such as 15,729 protein-coding genes, a 48.51% repetitive content, and syntenic relationships (including chromosomal fusion/fission events) with the well-studied domestic silkworm (*Bombyx mori*). We further discovered millions of genetic variations, including SNPs, InDels, and SVs. All the data are freely available in the SilkMeta database, helping researchers and breeders improve silk production, explore sustainable food sources, and advance research on insect biology.

## 1. Introduction

The eri silkworm *Samia cynthia ricini* (*S. ricini*, Lepidoptera: Saturniidae), domesticated from *Samia canningi*, was initially reared in northeastern India for silk production and later introduced to regions including China, Japan, Korea, and Europe [1]. Eri silkworm pupae are rich in protein, serving as a valuable nutritional resource for human consumption and as feed in aquaculture and poultry industries [2,3,4], making this species economically promising. Furthermore, as a newly established lepidopteran model, the eri silkworm exhibits biological properties such as polyphagy, multivoltinism, colored larval epidermis, disease resistance, and a ZZ/Z0 sex-determination system (2n = 28♂/27♀, ZZ♂/Z0♀), which are of significant interest to insect biologists [1,5,6]. These characteristics highlight its dual importance for advancing sericulture practices and deciphering fundamental mechanisms in insect biology.

Over the past two decades, genomic advancements have revolutionized functional genomics and breeding technologies in crops and livestock. For the mulberry silkworm, *Bombyx mori* (*B. mori*), a close relative of *S. ricini*, genome assemblies were released and updated in 2004, 2008, and 2019 [7,8,9], while genetic variation maps were constructed and refined in 2009, 2018, and 2022 [10,11,12]. These resources have enabled breakthroughs in various fields, allowing researchers to decipher the genetic basis of domestication history [11,12], behavior degenerations [13,14], and phenotype mutations [15]; identify targets for breeding traits like silk yield [12,16,17] and disease resistance [18,19]; and elucidate genes governing growth and development (e.g., moultinism and sexual regulation) [20,21]. In 2022, a comprehensive silkworm pan-genome further empowered high-throughput allele mining for functional genomics and breeding [12]. However, aside from the draft genome published in 2021 [5], genomic advancements for *S. ricini* remain limited. Notably absent is a chromosome-level assembly, which is critical for studying chromosomal evolution and precise genomic variation identification.

Here, we employed a hybrid assembly strategy integrating long-read, short-read, and Hi-C scaffolding, coupled with a comprehensive annotation pipeline using five major public databases (NR, SwissProt, Pfam, GO, and KEGG) and synteny analysis with *B. mori*. Protein-coding genes were predicted through integrated homology-based and transcriptome-evidenced approaches. The synteny analysis revealed chromosomal fusion/fission events and inversions between *S. ricini* and *B. mori*. Leveraging this assembly alongside existing sequencing data, we systematically identified genomic variations, including single nucleotide polymorphisms (SNPs), short insertions/deletions (InDels; <50 bp), and structural variations (SVs; >50 bp). All data were integrated into the SilkMeta database (http://silkmeta.org.cn (accessed on 15 April 2025)) for public access. These resources will allow for accelerated eri silkworm breeding and functional genomics while providing a high-quality reference genome for evolutionary and functional genomic studies across Lepidoptera.

## 2. Materials and Methods

### 2.1. Genome, Transcriptome, and Hi-C Sequencing

The experimental *S. ricini* specimens (GX) were provided by the Guangxi Institute of Sericulture Science. Larvae were reared on *Ricinus communis* leaves under controlled conditions: 27–29 °C with 80–90% relative humidity for the first to third instar, and 25–27 °C with 75–80% relative humidity thereafter in an air-conditioned room at Guangxi Institute of Sericulture Science, Nanning, China (22°83′ N, 108°31′ E).

For long-read sequencing, we prepared Oxford Nanopore Technology (ONT) libraries using genomic DNA extracted from a single female (GX-F) and a single male (GX-M) pupa, optimized for 20 kb fragment sizes. Library preparation targeted 9 μg DNA input, followed by sequencing on the PromethION platform (Oxford Nanopore Technologies, Oxford, UK) using R9.4 flow cells with SQK-LSK109 chemistry (Oxford Nanopore Technologies, Oxford, UK). For short-read sequencing, paired-end libraries with 300–400 bp insert sizes were constructed and sequenced on the DNBSEQ platform (MGI Tech, Shenzhen, China), using 1 μg genomic DNA for library construction. Raw data from both platforms were processed according to established protocols described in reference [12].

For RNA-seq, total RNA was extracted separately from larval, pupal, and adult stages using Trizol reagent (Simgen, Hangzhou, China). Equimolar RNA pools from each developmental phase were combined to construct a normalized composite library. The concentration and purity of pooled RNA were quantified using a Nanodrop 2000c spectrophotometer (Thermo Fisher Scientific, Waltham, MA, USA), while RNA integrity was evaluated by agarose gel electrophoresis. The sequencing library was constructed using the VAHTS Universal V10 RNA-seq Kit (Cat: NR616-02, Lot: 7E831C4, Vazyme, Nanjing, China) with 1 μg of total RNA, targeting an insert size of approximately 300 bp. Sequencing was performed on the DNBSEQ-T7 platform (MGI Tech, Shenzhen, China).

For Hi-C sequencing, pupal tissue (GX-M) underwent formaldehyde crosslinking followed by MboI restriction digestion. Biotinylated restriction fragments were ligated using T4 DNA ligase, then reverse crosslinked through sodium dodecyl sulfate (SDS) and protease K treatment. Streptavidin magnetic bead enrichment captured junction fragments, which were processed through end repair, adapter ligation, and PCR amplification. The final library was constructed using 500 ng of genomic DNA, followed by sequencing on the DNBSEQ system (MGI Tech, Shenzhen, China).

### 2.2. Genome Assembly

Prior to assembly, k-mer frequency analysis (k = 17) was performed using Jellyfish v2.2.6 [22]. Genome characteristics, including estimated size, repeat content, and heterozygosity rate, were subsequently predicted through genomeScope v1.0 [23] analysis using short-read sequencing data.

The de novo genome assembly was conducted as described in a previous report [12]. Briefly, raw ONT reads were error-corrected using Canu v1.8 [24], and the corrected reads were assembled into contigs using Smartdenovo v1.0 [25]. The contigs underwent three rounds of polishing with Racon v1.3.3 [26], followed by a final polishing step with Medaka v0.7.1 (https://github.com/nanoporetech/medaka (accessed on 20 July 2020)). Using this pipeline, genome assemblies for both GX-M and GX-F were generated. Chromosome-level scaffolding of the GX-M genome was achieved by integrating Hi-C data with the 3D-DNA pipeline [27]. The GX-F genome assembly was not scaffolded using Hi-C technology.

A previous study has established that *S. ricini* telomeres consist of (TTAGG)_n_ repeats [28]. Using tidk v0.2.63 [29] with parameter -w 100000, we identified terminal (TTAGG)_n_ motifs. If a chromosome exhibits more than 300 TTAGG repeats at its termini, it is considered as containing telomeric sequences.

For final assembly validation, we performed two complementary analyses:

(a) All short-read sequencing data were aligned to the assembled genome to calculate genome-wide coverage and mapping efficiency (considering all successfully mapped reads).
(b) Genome integrity was evaluated using Benchmarking Universal Single-Copy Orthologs (BUSCO) v5.5.0 [30] with the Lepidoptera_odb10 database, which reports percentages of complete single-copy, duplicated, and fragmented orthologs.

The chromosome-level GX-M genome was used as the reference for subsequent annotation and variation calling pipelines.

### 2.3. Genome Annotation

Repeat sequences in the genome were annotated through de novo prediction approach. A custom repeat library was constructed using RepeatModeler v2.0.1 (http://www.repeatmasker.org/RepeatModeler/ (accessed on 1 November 2024)) with the -LTRStruct parameter, followed by repeat identification and masking through RepeatMasker v4.1.0 (http://repeatmasker.org/ (accessed on 4 November 2024)).

Protein-coding gene prediction combined homology-based and transcriptome-based methods. For homology prediction, we curated protein sequences of *Manduca sexta*, *Chilo suppressalis*, *Papilio Xuthus*, *Spodoptera frugiperda*, and *Bombyx mori* from NCBI and SilkMeta (http://silkmeta.org.cn (accessed on 12 March 2025)) to build a reference protein database [31]. The database was analyzed alongside Braker3 v3.0.8 [32] for gene prediction. For transcriptome evidence, RNA-seq reads were aligned to the genome using STAR v2.7.11b [33] to generate a sorted BAM file, which was subsequently processed with Braker3 (default parameter). Predictions from both approaches were consolidated into a non-redundant gene set via TSEBRA v1.1.2.5 (default parameter) [32]. Subsequently, the integrated gene set underwent comprehensive validation using BUSCO analysis against the Lepidoptera_odb10 database to assess genome annotation completeness.

Functional annotation involved three complementary strategies:

(a) Sequence homology: Diamond v2.1.10 [34] was used for NR and SwissProt database searches.
(b) Domain identification: Pfam domains were annotated using InterProScan v5.72-103.0 [35].
(c) Pathway mapping: Gene Ontology (GO) terms and Kyoto Encyclopedia of Genes and Genomes (KEGG) pathways were assigned using the eggNOG-mapper v2.1.12 (http://eggnog-mapper.embl.de (accessed on 14 March 2025)) online tool.

Transfer RNAs (tRNAs) were identified using tRNAscan-SE v2.0.12 [36], ribosomal RNAs (rRNA) were annotated with rnammer v1.2 [37], and other non-coding RNA (ncRNA), including microRNAs (miRNAs) and small nuclear RNAs (snRNAs), were annotated by searching against the Rfam database (http://rfam.xfam.org/ (accessed on 9 May 2025)) using Infernal v1.1.5 [38].

### 2.4. Collinearity Analysis

*Bombyx mori* (2n = 56 chromosomes), a phylogenetically close relative of *S. ricini* (2n = 28), served as the reference for comparative karyotype analysis. To investigate the chromosomal evolution patterns of *S. ricini* GX-M and *B. mori* Dazao, we conducted a whole-genome synteny analysis using JCVI v1.4.16 [39] with the default parameters. The coding DNA sequences (CDS) of both species were input into JCVI, and the parameter -m jcvi.graphics.karyotype was applied to generate synteny visualization plots for comparative genomic analysis.

### 2.5. Identification and Annotation of SNPs, InDels, and SVs

We identified SNPs and InDels using short-read sequencing data and detected SVs using long-read sequencing data. In addition to our two *S. ricini* samples (GX-M and GX-F), we incorporated publicly available short-read and long-read sequencing data of a Japanese *S. ricini* strain (UT; NCBI accession GCA_014132275.2). Short-read data from GX-F and UT were mapped to the GX-M reference genome using BWA v0.7.17 [40] with default parameters. Unmapped and duplicated reads were filtered using SAMtools v1.9 [41] and Picard v2.18.29 (https://broadinstitute.github.io/picard/ (accessed on 22 March 2025)). Raw SNP/InDel variants were called through GATK4 v4.4.0.0 [42]. The HaplotypeCaller generated GVCFs, followed by CombineGVCFs and GenotypeGVCFs for joint genotyping.

Variant filtering was performed using GATK v4.4.0.0 VariantFiltration with stringent thresholds. The SNPs were filtered with the parameters “QD < 2.0, QUAL < 30.0, SOR > 3.0, FS > 60.0, MQ < 40.0, MQRankSum < −12.5, ReadPosRankSum < −8.0”, while InDels were filtered with the parameters “QD < 2.0, QUAL < 30.0, FS > 200.0, ReadPosRankSum < −20.0”.

For SV detection, long-read data from both *S. ricini* specimens were mapped to the GX-M genome using NGMLR v0.2.7 [43], followed by SV calling, merging, and filtering with the combined calling method (default parameters) of Sniffles2 v2.2 [44]. The combined calling method integrates multi-sample SV detection, merging, and automated filtering into a unified workflow, directly generating a high-quality SV set as the final output. All variants were functionally annotated for gene/variant positional relationship using SnpEff 4.3t [45].

## 3. Results

### 3.1. Genome Sequencing and Assembly

One male (GX-M) and one female (GX-F) *S. ricini* were sequenced using the DNBSEQ platform (short read), the Oxford Nanopore system (long read), and Hi-C technology. The short-read sequencing of *S. ricini* GX-M and GX-F samples generated 31.88 Gb (70×) and 41.97 Gb (93×) of data, respectively (Table 1). Long-read ONT sequencing for these samples produced 65.27 Gb (145×) and 63.16 Gb (140×) of data, with read N50 values of 18,508 bp and 21,955 bp for GX-M and GX-F, respectively (Table 1). Notably, the maximum read lengths achieved were 155,012 bp (GX-M) and 157,443 bp (GX-F). Hi-C sequencing produced 47.80 Gb of chromatin interaction data from the male specimen (Table 1).

**Figure 1:**
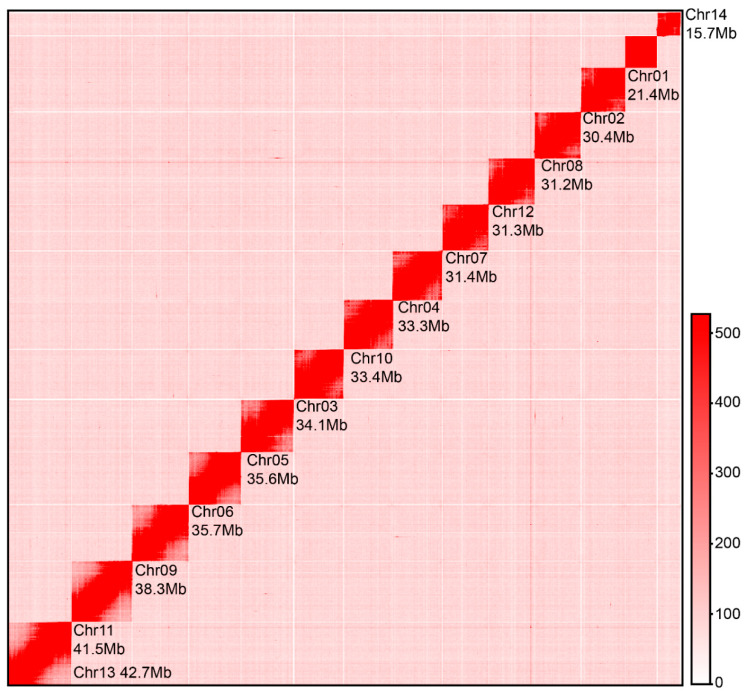
Hi-C chromosomal contact map of *S. ricini*. The interaction map shows a clear structural configuration of the 14 *S. ricini* chromosomes, characterized by strong intra-chromosomal interactions and low inter-chromosomal signal noise, which underscores the high resolution of chromosome architecture.

Genome survey analysis based on short-read data estimated a genome size of 451.05 Mb (GX-M) and 438.25 Mb (GX-F), with repetitive sequence contents of 47.68% and 47.49%, and heterozygosity rates of 0.25% and 0.36%, respectively. Preliminary assemblies generated 73 contigs (N50 = 18.55 Mb, total size = 457.85 Mb) for the GX-M genome and 63 contigs (N50 = 25.31 Mb, total size = 455.45 Mb) for the GX-F genome. Hi-C scaffolding enabled the chromosomal-level assembly of the GX-M genome, anchoring 59 contigs into 14 chromosomes with a final size of 456.16 Mb (Table 2 and Table 3, Figure 1). A total of 11 telomeric sequences were detected across 14 chromosomes, with 3 of these chromosomes showing telomeres at both ends (Table 3).

To assess genome assembly quality, we mapped short-read sequencing data to GX-M and GX-F *S. ricini* genomes independently. The read-mapping ratios reached 99.86% and 99.65%, with genome coverage values of 99.61% and 99.65%, respectively. BUSCO analysis revealed 98.5% completeness for both genomes. The GX-M assembly contained 98.1% complete and single-copy BUSCOs and 0.4% complete and duplicated BUSCOs, while the GX-F assembly showed 98.2% complete and single-copy BUSCOs and 0.3% complete and duplicated BUSCOs. These metrics confirmed the high completeness and reliability of the assembled genomes.

### 3.2. Genome Annotation

The *S. ricini* genome contained 222.09 Mb of repeat sequences, representing 48.51% of the total assembly. Transposable elements (TEs) dominated the repeat landscape, constituting 47.22% of the genome. TE composition analysis revealed distinct class distributions: long interspersed nuclear elements (LINEs) were most prevalent (18.97%), followed by helitrons (8.81%), unclassified elements (13.53%), DNA transposons (2.73%), long-terminal repeat (LTR) elements (2.54%), and short interspersed nuclear elements (SINEs) at 0.64% (Table 4).

**Figure 2:**
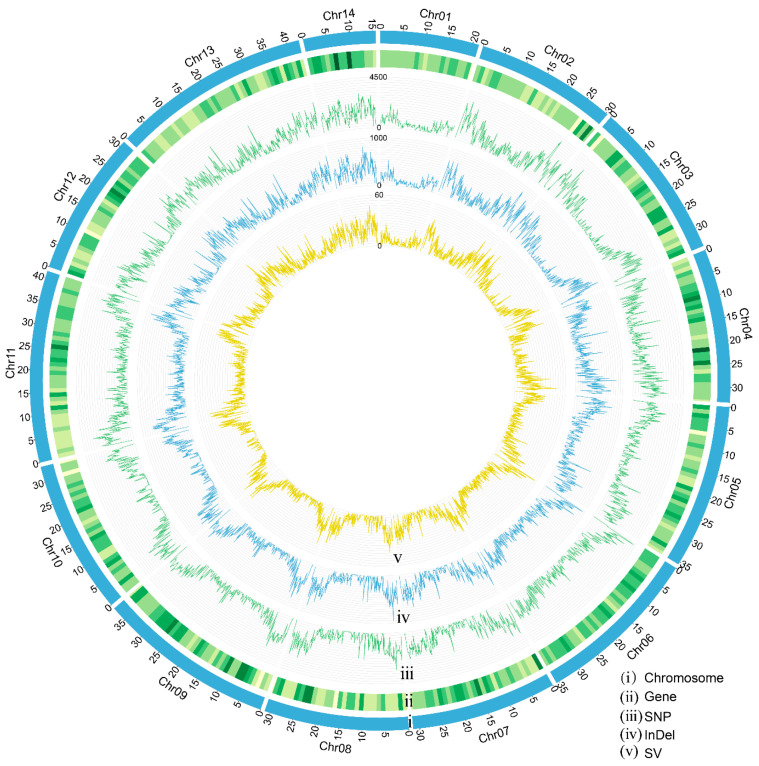
Chromosomal distribution of protein-coding genes and genetic variations in the *S. ricini* genome. (i) Chromosome sizes of *S. ricini*. (ii) Density of genes, (iii) SNPs, (iv) InDels, and (v) SVs across all *S. ricini* chromosomes.

**Figure 3:**
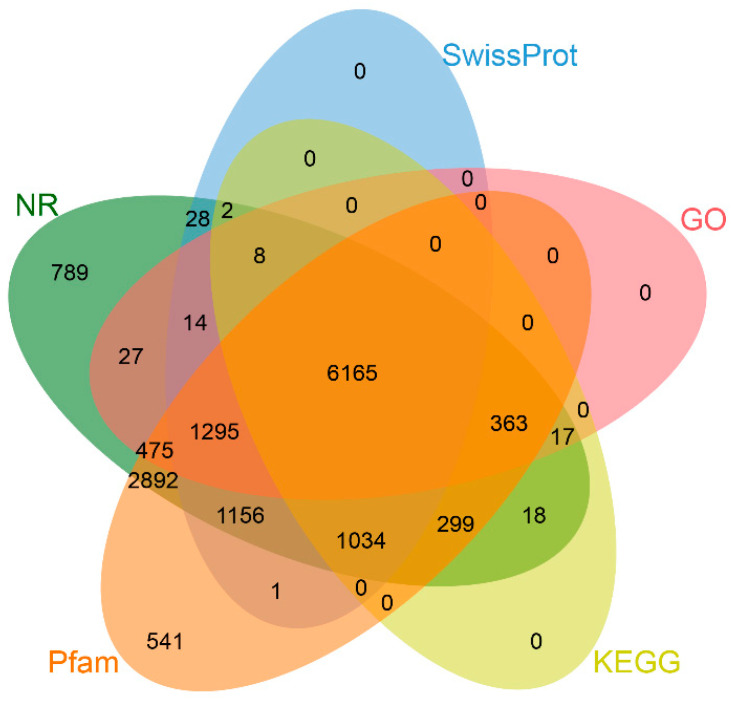
Integrated functional annotation of *S. ricini* protein-coding genes across five databases (NR, Swiss-Prot, Pfam, Gene Ontology, and KEGG pathways).

A total of 15,729 protein-coding genes were predicted in the GX-M genome through integrated RNA- and homology-based annotation approaches (Figure 2). Furthermore, we identified 1175 tRNAs, 112 rRNAs, and 466 other ncRNAs within this genome (Table S1). BUSCO analysis against the Lepidoptera_odb10 database demonstrated 98.2% completeness, comprising 96.6% complete single-copy and 1.6% complete duplicated orthologs, confirming high gene prediction accuracy. Functional annotation through database searches yielded the following results: 14,582 genes (92.71%) matched to NR, 9703 (60.91%) to SwissProt, 14,221 (90.41%) to Pfam, 8364 (53.18%) to GO, and 7906 (50.26%) to KEGG pathways. Cross-database analysis identified 6165 genes (39.2% of the total) annotated across all five databases (Figure 3).

### 3.3. Collinearity with Bombyx Mori Genome

**Figure 4:**
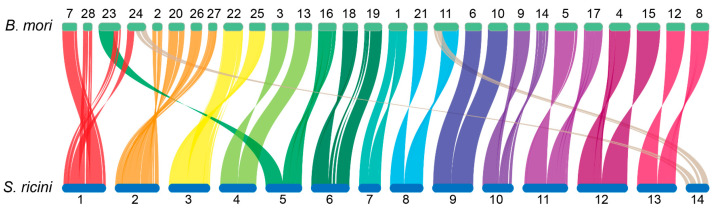
Collinearity of *S. ricini* and *B. mori* genomes.

*Bombyx mori*, a lepidopteran model organism with 28 (2n = 56) chromosomes, served as the reference for comparative genomic analysis. Syntenic relationship analysis between *S. ricini* and *B. mori* genomes revealed strong collinearity, accompanied by chromosomal fusion/fission events and inversions. The syntenic relationships between the two lepidopteran genomes were characterized by the following chromosomal correspondences:

*Bombyx mori* Chr7, partial Chr23/24, and Chr28 aligned with *S. ricini* Chr1, while Chr2/20/26/27 corresponded to *S. ricini* Chr2. Fusion events were also observed between *B. mori* Chr22/25 and *S. ricini* Chr3, as well as between *B. mori* Chr3/13 and *S. ricini* Chr4. Partial Chr23 and Chr16 of *B. mori* showed synteny with both *S. ricini* Chr5. Notably, *B. mori* Chr1 exhibited synteny with *S. ricini* Chr7 with a large-scale inversion. Additional syntenic blocks included the following: *B. mori* Chr18/19 with *S. ricini* Chr6; Chr11/21 with Chr8; Chr6/10 with Chr9; Chr9/14 with Chr10; Chr5/17 with Chr11; Chr4/15 with Chr12; and Chr8/12 with Chr13. A secondary syntenic association was identified between partial *B. mori* Chr11/24 and *S. ricini* Chr14 (Figure 4).

### 3.4. Variation Map

An analysis of short-read and long-read data identified 1,771,512 SNPs (raw count: 1,868,658) and 433,440 InDels (raw count: 470,271) in the GX-F sample, alongside 25,567 SVs. For the UT strain, we detected 3,362,499 SNPs (raw count: 3,551,595), 780,961 InDels (raw count: 818,341), and 48,757 SVs. Through combined variant calling, we established a non-redundant variation set totaling 4,270,848 SNPs (raw count: 4,509,966), 1,021,705 InDels (raw count: 1,066,653), and 53,367 SVs (Table 5).

The chromosomes of *S. ricini* displayed distinct spatial distributions of genomic features, including genes, SNPs, InDels, and SVs (Figure 2). Notably, chromosome 1 exhibited relatively lower genetic variation frequencies and gene density compared to other chromosomes. Furthermore, variation density was significantly higher in the terminal regions of chromosomes than in the central regions, a distribution pattern consistent with the genomic architecture of *B. mori* [12].

SNP distribution analysis revealed that 1,953,178 (45.63%) were in intergenic regions, 1,197,685 (27.98%) were in introns, 429,159 (10.03%) were downstream, 596,161 (13.93%) were upstream, and 104,631 (2.45%) were in exons (Table 5). Among exonic SNPs, 62,880 (60.10%) were synonymous substitutions, 41,751 (39.90%) were non-synonymous mutations, and 361 introduced premature stop codons (Table 5). The SNP density across the whole genome is 107 bp/SNP, while in exons, introns, and intergenic regions, the densities are 369 bp/SNP, 179 bp/SNP, and 70 bp/SNP, respectively (Table 6).

InDel analysis revealed near-equal proportions of insertions (486,695; 47.64%) and deletions (535,010; 52.36%). Genomic distribution included 445,118 (43.46%) intergenic, 316,562 (30.91%) intronic, 142,907 (13.95%) downstream, 114,744 (11.20%) upstream, and 4933 (0.48%) exonic variants (Table 5). Functional impacts included 1798 (0.11%) frameshifts and 158 (0.01%) start/stop codon alterations.

**Figure 5:**
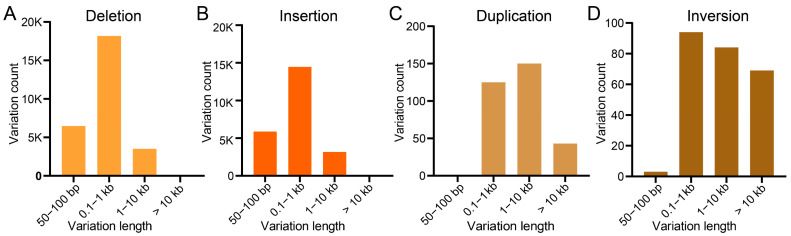
Lengths and counts of (**A**) deletions, (**B**) insertions, (**C**) duplications, and (**D**) inversions in the *S. ricini* genome.

SV characterization identified 23,602 insertions, 28,358 deletions, 318 duplications, and 250 inversions. predominantly 100 bp-1 kb in length (Figure 5A–D). Distribution patterns showed 22,687 (41.19%) intergenic, 15,960 (28.98%) intronic, 7434 (9.75%) upstream, 5249 (9.53%) downstream, and 3746 (6.80%) exonic SVs (Table 5). Functional consequences included frameshifts (817, 1.48%), stop codon gains (388, 0.70%), and exon losses (96, 0.17%). Analysis of SV length distributions demonstrated that the majority of insertions and deletions range from 100 bp to 1 kb (Figure 5A,B), while most duplications and inversions span 100 bp to 10 kb (Figure 5C,D).

### 3.5. Visit Samia Ricini Genome

**Figure 6:**
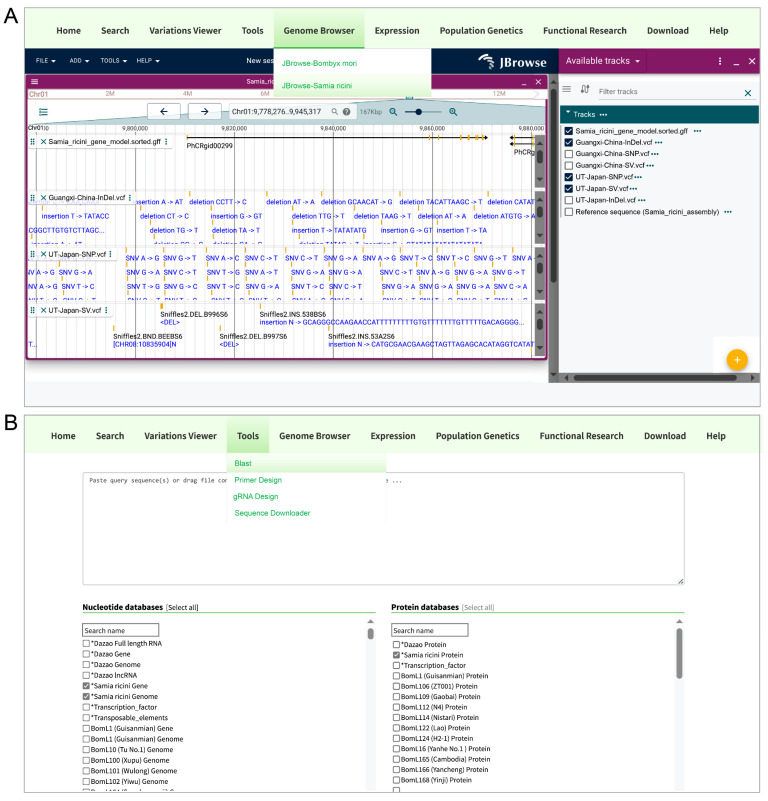
SilkMeta database interface for *S. ricini* genomic exploration. (**A**) Genome browser. Interactive visualization of *S. ricini* chromosomal assembly, annotated gene models, and genomic variations (SNPs/InDels/SVs). (**B**) BLAST module. Sequence homology search interface for querying *S. ricini* genomic DNA, coding sequences (CDSs), and protein datasets. The asterisks (*) are used to move the marked items to the top position.

To enhance the accessibility of *S. ricini* genomic resources, we integrated the assembled genome and variant datasets (SNPs, InDels, SVs) into the SilkMeta database (http://silkmeta.org.cn (accessed on 15 April 2025)) through three functional modules: Genome browser, BLAST v2.14.0+, and Download. The genome browser allows for interactive visualization of the chromosome-level assembly, annotated gene models, and genomic variations (SNPs/InDels/SVs) via a user-friendly interface (Figure 6A). The BLAST tool enables sequence similarity searches against *S. ricini* genomic, coding (CDS), and protein sequences through customizable query parameters (Figure 6B). The Download module offers comprehensive data downloads, including genome assembly (FASTA), gene annotations (GFF), coding/protein sequences, and variant call format (VCF) files. This integration facilitates seamless exploration, analysis, and utilization of *S. ricini* genomic data for the research community.

## 4. Discussion

The *S. ricini* is a key resource insect of agricultural and biological importance. This study delivers a chromosome-scale genome and variation map for *S. ricini*, addressing gaps in lepidopteran genomics. Using Hi-C sequencing data, the 456.16 Mb assembly was anchored to 14 chromosomes, which corresponded to a previous karyotype analysis [46]. The chromosome-level genome assembly and gene annotation of the *S. ricini* presented in this study demonstrate higher completeness and integrity than previous genomic resources for this species (Table 7). Moreover, the assembly quality surpasses most existing genomic datasets from other Bombycidae (silkworm) and Saturniidae (giant silkworm moth) species (Table 7).

The correct assembly of chromosomes is an essential foundation for us to conduct synteny analysis and accurately identify chromosomal rearrangement events. Our synteny analysis with *B. mori* revealed conserved macrosyntenic blocks interspersed with chromosomal rearrangements. These rearrangements were predominantly driven by fusion/fission events, with only a single intrachromosomal inversion detected on chromosome 7 of *S. ricini* (orthologous to *B. mori* chromosome 1). This conserved syntenic architecture aligns with a prior cytogenetic mapping study in these species [46] and further supports the internal stability of lepidopteran chromosomes. Future studies leveraging these high-quality genomes should elucidate precise mechanisms of chromosome fusion/fission between *S. ricini* and *B. mori*, including gene order conservation within syntenic blocks and structural features at rearrangement breakpoints.

Genomic variations are valuable for functional genomic analysis and molecular marker-assisted breeding. Previously, Simple Sequence Repeat (SSR) markers of *S. ricini* were developed to evaluate genetic diversity, adaptive evolution, and trait-associated genes [53,54,55]. The genomic variations identified in this study establish a foundational resource for eri silkworm research. These variants serve as standardized genomic markers for establishing phenotype-genotype associations across populations. Furthermore, they constitute high-value markers for designing high-density genotyping arrays, essential tools for cost-efficient population genetics analyses, quantitative trait locus (QTL) mapping, genome-wide association studies (GWAS), and genomic selection breeding programs. Implementation of these resources will accelerate research on the genetic mechanisms underlying agronomically important traits (e.g., disease resistance and silk yield) and enhance marker-assisted breeding in the eri silkworm. We acknowledge that variant discovery from limited samples carries inherent constraints for population-level inferences. Comprehensive characterization of genomic variation across hundreds of individuals remains a critical objective for future studies.

Genomic data sharing remains a major focus for biologists, yet significant limitations persist in the visualization, access, and analysis of the *S. ricini* genome. Leveraging SilkMeta [31], a robust pan-genome and multi-omics database for *B. mori*, we implemented its data-sharing and analytical framework to enable interactive visualization of the *S. ricini* genome and variation datasets, providing researchers with an intuitive interface for genomic exploration. This initiative represents a critical strategy to maximize the utility of *S. ricini* genomic resources in comparative studies of eri silkworms and broader insect genomics.

In summary, the chromosome-level genome assembly, comprehensive variation dataset, and publicly accessible platform established in this study form an essential foundation for advancing comparative genomic research in *S. ricini* and related insect species.

## 5. Conclusions

This study presents a chromosome-level genome assembly of *Samia cynthia ricini* (456.16 Mb, scaffolded onto 14 chromosomes) generated using integrated long-read, short-read, and Hi-C sequencing data. The assembly achieved 98.5% BUSCO completeness and contains 15,729 predicted protein-coding genes. Functional annotation against five major databases (NR, SwissProt, Pfam, GO, and KEGG) revealed a maximum annotation rate of 92.71% (NR). Comparative genomics with *Bombyx mori* uncovered conserved syntenic blocks interspersed with chromosomal fusion/fission events and an intrachromosomal inversion. Furthermore, we established a comprehensive variation map, identifying 4.27 million SNPs, 1.02 million InDels, and 53,367 SVs, serving as critical resources for trait association studies and molecular breeding. All data are freely accessible via the SilkMeta database (http://silkmeta.org.cn (accessed on 15 April 2025)), providing an essential platform for sustainable utilization of this agriculturally significant insect.

## Figures and Tables

**Table 1:** Summary of *S. ricini* genome sequencing data.

Sample	Sequencing Platform (Technology)	Clean Reads	Clean Bases (Gb)	Genome Coverage (×)	Reads N50 Length (bp)
GX-M	DNBSEQ	216,133,152	31.88	70	-
	ONT	4,028,575	65.27	145	18,508
	Hi-C	319,016,548	47.80	-	-
GX-F	DNBSEQ	283,461,080	41.97	93	-
	ONT	3,476,160	63.16	140	21,955

**Table 2:** Summary of genome assembly.

Sample	Initial Assembly			Hi-C Assembly	
	Contigs	Contig N50 Length (bp)	Total Length (bp)	Contigs	Total Length (bp)
GX-M	73	18,557,025	457,852,231	59	456,164,652
GX-F	63	25,316,322	455,453,376	-	-

**Table 3:** Chromosome length and telomeres of the *S. ricini* genome.

Chromosome ID	Length (bp)	Telomere
Chr01	21,357,909	0
Chr02	30,424,992	1
Chr03	34,141,321	1
Chr04	33,297,754	1
Chr05	35,620,675	0
Chr06	35,677,325	0
Chr07	31,408,718	0
Chr08	31,248,863	1
Chr09	38,345,835	2
Chr10	33,366,736	0
Chr11	41,493,536	2
Chr12	31,340,067	0
Chr13	42,746,998	2
Chr14	15,693,923	1
Total	456,164,652	11

Note: Values of 2, 1, and 0 in the Telomere column denote the number of telomeric regions identified in each of the 14 chromosomes of *S. ricini* genome (2, 1, or none detected). The total of 11 indicates all telomere regions identified across the genome.

**Table 4:** Statistics on repeat sequences in the *Samia ricini* genome.

Type of Repetitiveness		Length (bp)	Percentage in Genome (%)
TEs	SINEs	2,915,523	0.64
	LINES	86,866,339	18.97
	LTR	11,617,914	2.54
	Penelope	1,244,859	0.27
	DNA transposons	12,499,705	2.73
	Helitrons (rolling-circles)	40,349,215	8.81
	Unclassified	61,962,172	13.53
Small RNA		263,243	0.06
Simple repeats		4,853,247	1.06
Low complexity		819,131	0.18
Total		222,094,622	48.51

**Table 5:** Summary of variations within the *S. ricini* genome.

Variation Type	Variation Counts		Relative Positions to Protein-Coding Genes					
	Before Filtering	After Filtering	Intergenic	Intron	Down-Stream	Up-Stream	Exon (Synonymous)	Exon (Non-Synonymous)
SNP	4,509,966	4,270,848	1,953,178 (45.63%)	1,197,685 (27.98%)	429,159 (10.03%)	596,161 (13.93%)	62,880 (1.47%)	41,751 (0.98%)
InDel	1,066,653	1,021,705	445,118 (43.46%)	316,562 (30.91%)	142,907 (13.95%)	114,744 (11.20%)	4933 (0.48%)	
SV	-	53,367	22,687 (41.19%)	15,960 (28.98%)	5249 (9.53%)	7434 (13.50%)	3746 (6.80%)	

**Table 6:** SNP densities in different genome regions of *S. ricini*.

	Whole Genome	Exon	Intron	Intergenic
DNA length (bp)	457,689,390	34,975,315	214,843,885	207,870,190
SNP count	4,270,848	94,665	1,197,685	2,978,498
SNP density (bp/SNP)	107	369	179	70

**Table 7:** Genome assembly and annotation statistics of *S. ricini* and other Bombycidae and Saturniidae silkworms.

Family	Species	Genome Size (Mb)	Chr. Anchoring Strategy	Chr. Numbers	Contig N50 (Mb)	BUSCO (Assembly)	BUSCO (Gene Model)	Repetitive Elements (%, bp)	Publish Year
Saturnii-dae	*Samia ricini* (China, GX-M)	456.16	Hi-C	*N* = 14	18.56	98.5%	98.2%	48.5	This study
	*Samia ricini* (China, GX-F)	455.45	none	*N* = 14	25.32	98.5%	-	-	This study
	*Samia ricini* (Japan, UT)	450.48	Linkage analysis	*N* = 14	21.37	97.9%	91.9%	43.5	2021 [5]
	*Antheraea assamensis*	501.18	none	*N* = 15	0.68	98.0%	96.0%	49.0	2024 [47]
	*Antheraea pernyi*	726.37	Hi-C	*N* = 49	13.77	-	95.6%	60.7	2020 [48]
	*Antheraea yamamai*	656.00	none	*N* = 31	0.74	96.7%	-	37.3	2018 [49]
Bombyci-dae	*Trilocha varians*	353.84	Optical mapping	*N* = 26	13.28	98.7%	98.6%	-	2024 [50] 2025 [51]
	*Bombyx mandarina* (Japan)	419.60	Hi-C	*N* = 27	16.43	95.1%	94.5%	-	2025 [52]

## Data Availability

Long-read (ONT), short-read (DNBSEQ), and Hi-C sequencing data for GX-M and GX-F have been deposited in the Genome Sequence Archive (GSA; https://ngdc.cncb.ac.cn/gsa/ (accessed on 5 May 2025)) at the China National Center for Bioinformation (CNCB) under Project ID PRJCA039017 (Accession: CRA025435). The chromosome-level genome assembly, coding sequences (CDS), protein sequences, annotation files (GFF3 format), and genomic variations (VCF format) are publicly accessible through the SilkMeta database at http://silkmeta.org.cn/download (accessed on 15 April 2025). Previously published long-read and short-read sequencing data were retrieved from the NCBI Sequence Read Archive (SRA) under BioProject PRJNA699736.

## Supplementary Materials

The following supporting information can be downloaded at https://www.mdpi.com/article/10.3390/biology14060698/s1. Table S1: Predicted non-coding RNAs in the *Samia ricini* genome.
